# Supplementary material for: Risk factors for bovine rotavirus infection and genotyping of bovine rotavirus in diarrheic calves in Bangladesh
Source: PLoS One. 2022 Feb 25;17(2):e0264577. doi: 10.1371/journal.pone.0264577 (PMC8880881; doi:10.1371/journal.pone.0264577)
Supplement: S1 Raw images — (PDF) [file pone.0264577.s002.pdf]

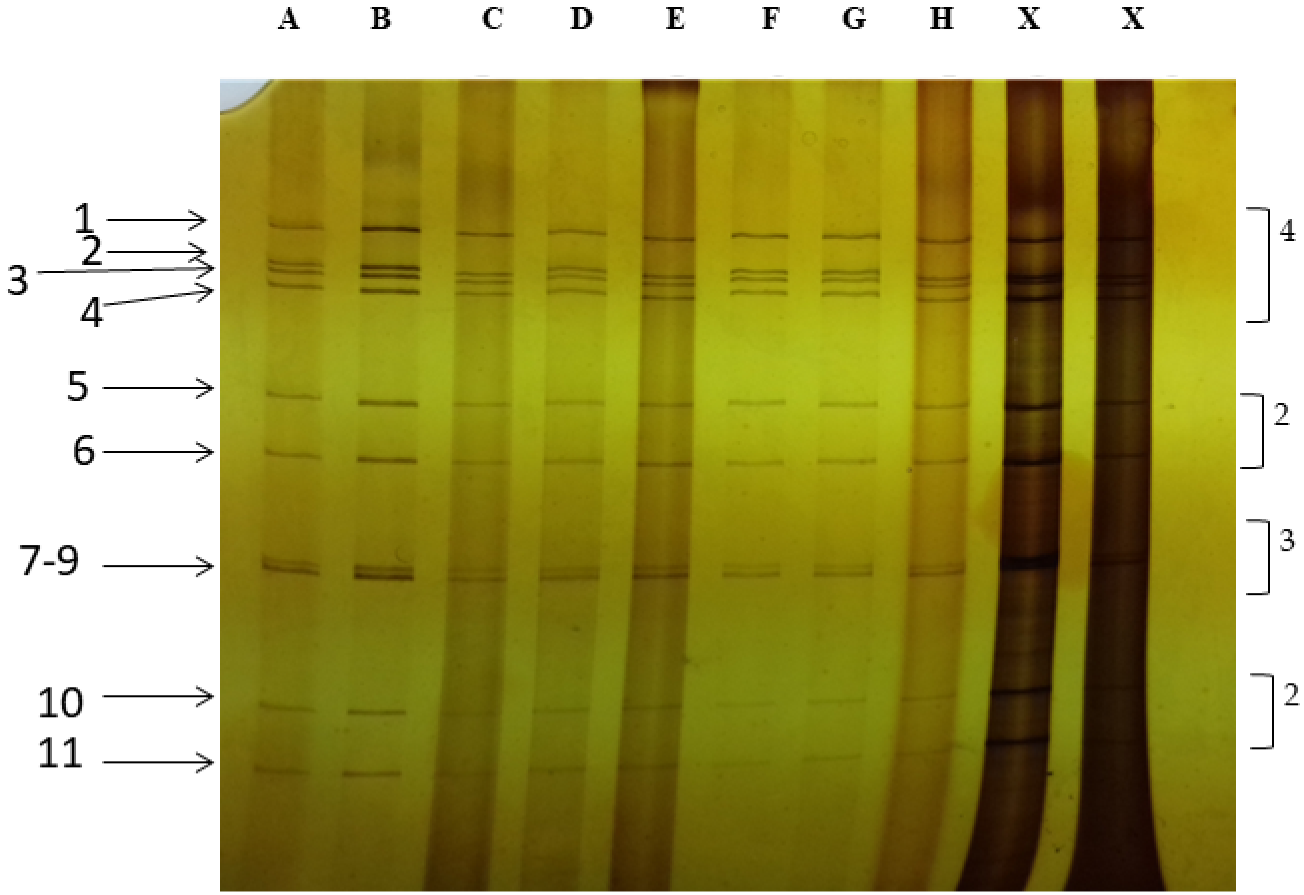

Fig. 1: Figure was captured by Gel documentation system (AlphaImager MINI, Cell Biosciences), lanes A-H were used to generate Figure 1.

(a)

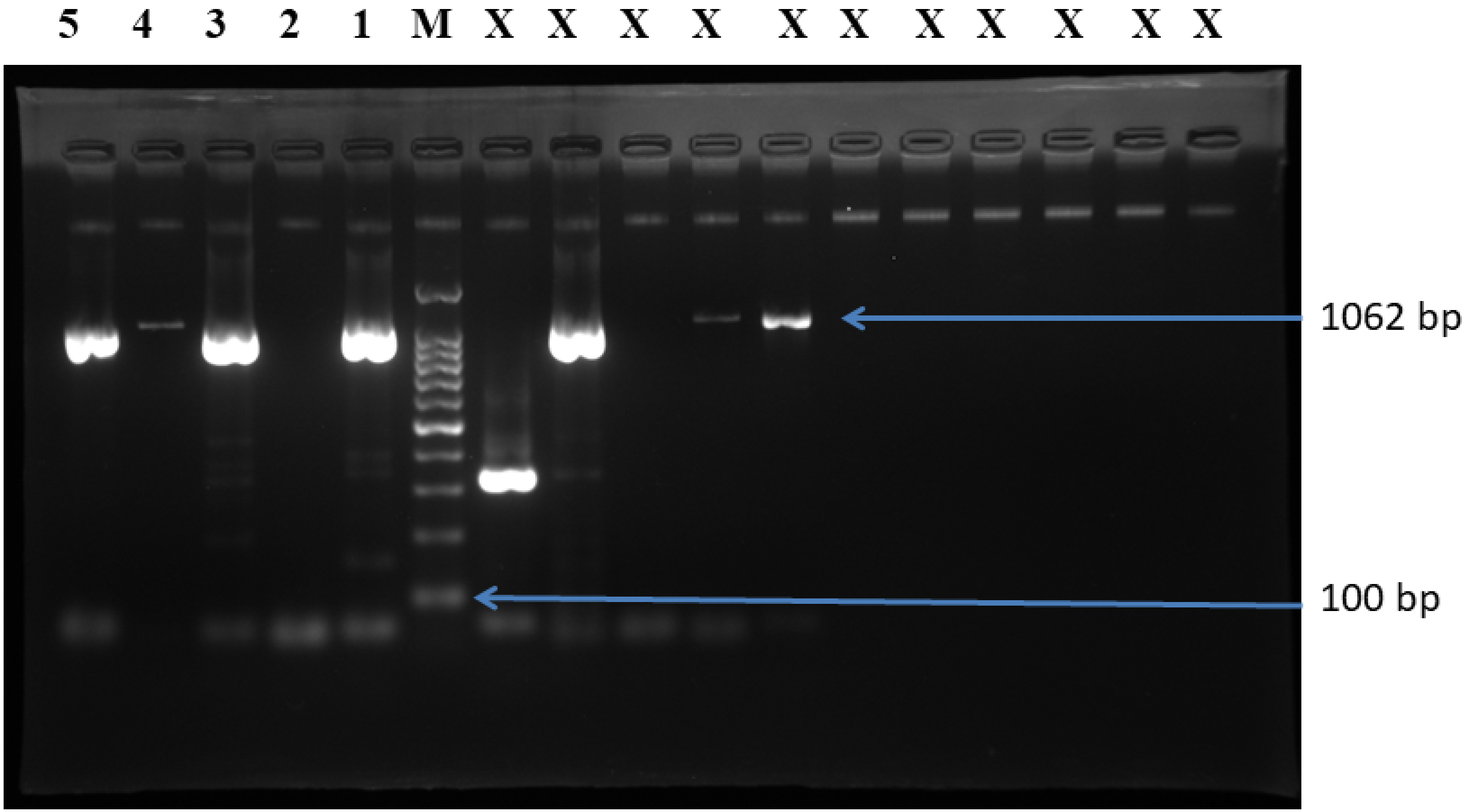

Fig. 2 (a): Figure was captured by Gel Documentation System (AlphaImager MINI, Cell Biosciences), Lanes M, 1, 2, 3, 4, and 5 were used to generate Figure 2 (a).

(b)

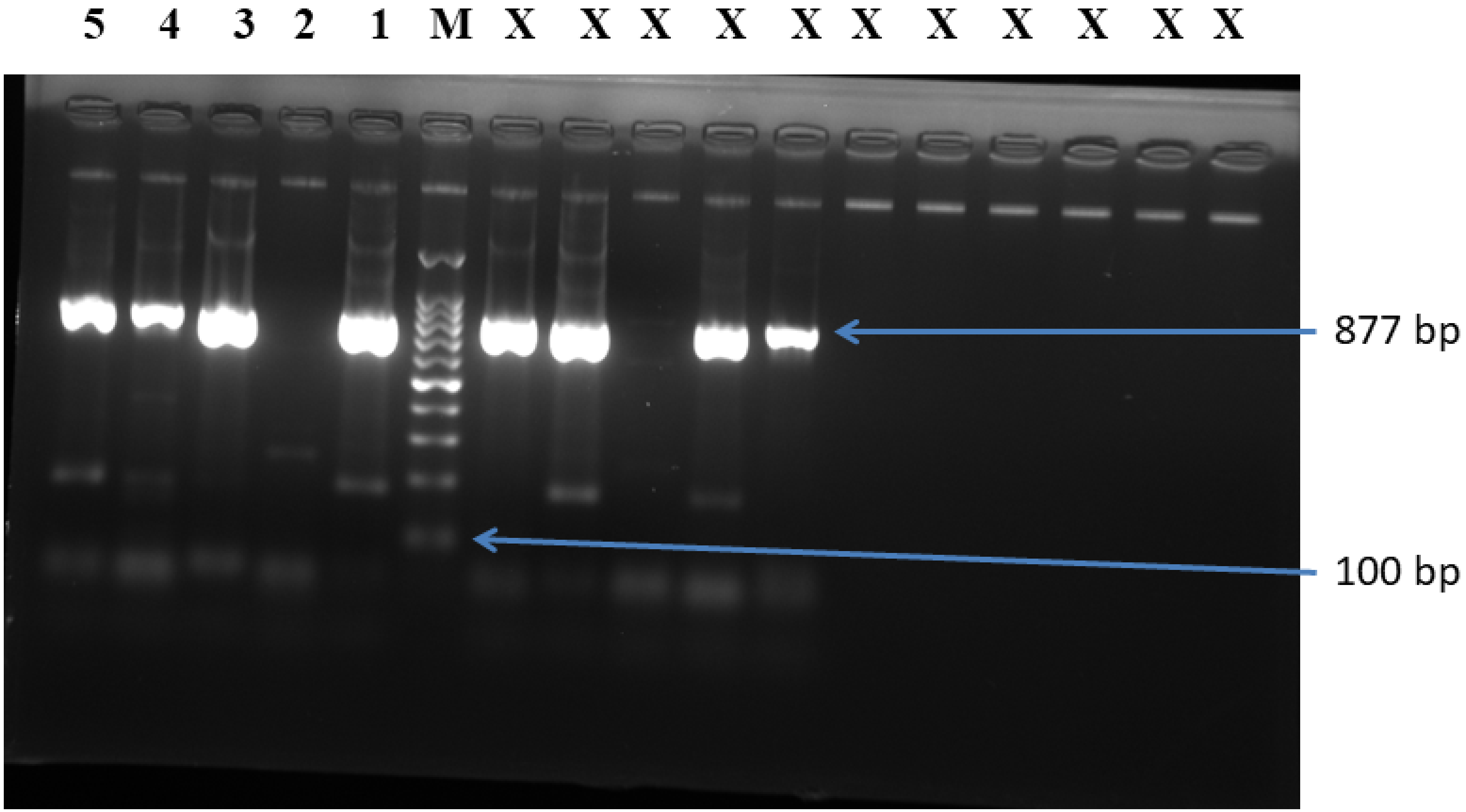

Fig. 2 (b): Figure was captured by Gel Documentation System (AlphaImager MINI, Cell Biosciences), Lanes M, 1, 2, 3, 4 and 5 was shown as Figure 2 (b).
