# Supplementary material for: Risk factors for bovine rotavirus infection and genotyping of bovine rotavirus in diarrheic calves in Bangladesh
Source: PLoS One. 2022 Feb 25;17(2):e0264577. doi: 10.1371/journal.pone.0264577 (PMC8880881; doi:10.1371/journal.pone.0264577)
Supplement: Attachment — Submitted filename: Response to Reviewers.docx [file pone.0264577.s004.docx]

We thank both reviewers for their valuable comments to improve the manuscript. The changes made in the revised manuscript were shown as blue font.

Reviewer #1: Minor comments

Abstract

1. The age of the calves should be preferably in weeks over months.

Response: The Age was categorized to weeks as suggested. Lines 36 and Tables 2 and 3.

2. Crossbred calves details like types, distribution among types, etc. missing.

Response: The details of crossbred calves have been given in Table 2.

3. Comparison to Indian strain of G6 and G10 w.r.t. specific locations in genotype should be elaborated.

Response: Elaborated as suggested by the reviewer. Lines 39-40.

Introduction

1. There are lot of lines where space between words are missing. For example, Line 50. "Bovinerotavirus" should be "Bovine rotavirus"; Line 100. "...,breedand location..." should be "..., breed and ..."; Line 108. "..placedin.." should be "...placed in...". Similarly for Line 116. 153. 162. 171. 200. 201. 202. 214. 226. 258. 304.

Response: Sorry for this unintentional mistake. They were corrected in the revised manuscript.

2. Line 52. RV is not abbreviated before.

Response: RV was elaborated at line 55.

3. The flow of introduction should be re-looked.

Response: The flow of the introduction was relooked.

4. Line 166. Details of Chromas 2.23 required.

Response: The details of the Chromas 2.23 were added in line 184.

5. Line 178. "ND: Note determined" should be "Not Determined"

Response: Corrected as Not Determined.

6. Table 3. Age should be presented in weeks instead of month. Breed details are required. Are Cross Breed unique or represent non-indigenous as a whole?

Response: Age was presented as weeks instead of months in the Table 3. The composition of cross breed calves was also provided in the Table 3.

7. Line 244. "This result...vice-versa" suggests transit of BRVs via established routes. Please revise the sentence appropriately.

Response: This sentence was revised as suggested by the reviewer. Lines: 267-268.

8. Line 224. "Blast" should be "BLAST"

Response: Blast was replaced by BLAST.

9. Line 314. "Legal and illegal...frequent". The author may substantiate this statement with reference.

Response: A reference was added to support this statement. Reference number 44.

10. What is the detection limit of the PCR-test used?

Response: We used conventional PCR so the detection limit is not applicable.

11. Different vaccines have been developed and deployed across the world. Serum Institute of India has made bovine rotavirus vaccine as well. The authors may comment upon the utility of known vaccines on the cohort tested.

Response: Serum Institute, India developed reasserted bovine pentavalent vaccine by using human G1, G2, G3, G4, and G9 serotypes in the genetic background of bovine rotavirus. This vaccine is intended for human use only. Although the name of the vaccine is bovine rotavirus pentavalent vaccine but principally this is not for animal use. We have mentioned in the submitted manuscript that the available vaccines having dominant genotypes in Bangladesh can also be used for our cattle population.

12. What is the vaccination status of the cohort and that of the herd from which the calves were selected? What criteria was used to determine selection of calves in the herd?

Response: Vaccination against rotavirus calf diarrhea has not yet initiated in Bangladesh. The calves were selected from the herd by a convenience sampling technique as we mentioned in the matherials and methods section. Line: 105.

13. How does the data correlate with Indian rotaviral reports like that of Tatte et a. 2019 (PMID: 30879319) and Kumar et al 2018 (PMID: 29464925), Chitambar 2011 (PMID: 20880637)?

Response: Tatte et al., reported 27.3% G10, while we detected only 3.3% G10. In addition the authors also reported P[6] and P[8] while we detected only p[11]. Similarly, Chitambar et al., 2011 reported G8P[14] which we did not detect. . Kumar et al., 2018 reported an unusual bovine rotavirus strain having similarly with human rotavirus which is not similar to our results. We have added this information in the discussion section.

14. Have the authors identified any similarities with the human rotaviruses?

Response: No, the bovine rotavirus genotypes we reported in this manuscript were not similar with human rotaviruses.

Reviewer #2: In the present study, Ahmed et al. has tried to identify G and P genotypes of bovine rotavirus (BRV) strains in diarrheic calves as well as the major risk factors of BRV infection. In this study, Ahmed et al. noticed the predominant role of G6P[11] genotype (94.4%), followed by G10P[11] (5.6 %) in BRV infection. Further, their experimental evidence suggested age and breed of claves as major risk factors for the BRV infection.

Comments:

1. Authors should also discuss the impact of other factors particularly, season and sex in the abstract.

Response: Season and sex were not significantly associated with BRV infection. So we did not add them in the abstract.

2. In the introduction section, authors have discussed more about the genotypes of BRV; it will be good, if they will reduce this part and include a paragraph on the impact of different parameters (such as Herd size, colostrum feeding, colostrum timing and colostrum amount) on the rate of BRV infection in calves.

Response: We have reduced the introduction section in relation to the BRV genotypes and added one paragraph about the effect of herd size, colostrum feeding, timing and amount. Lines: 87-94.

3. If possible, authors should also examine the impact of other factors on BRV infection, which are also identified as one of the potential factors (such as Herd size, colostrum feeding, colostrum timing and colostrum amount) for BRV infection.

Response: Thank you very much for this comment. It could be valuable if these variables were included in the analysis. However, we don’t have data for these variables.

4. In this study, author found that female calves are more susceptible for BRV infection while studies have also shown that male calves are more susceptible for BRV infection, what could be the possible reason; please discuss this in to the discussion section.

Response: As mentioned in the response to a previous comment of the reviewer that sex was not statistically associated with BRV infection. So we did not describe it in the manuscript.

6. Discuss the principle of rapid detection kit BIO K 152 behind the detection of BRV in the materials and methods.

Response: The principle of rapid detection kit BIO K 152 was added in the materials and method section as suggested by the reviewer. Lines: 113-116.

7. Authors should elaborate the methods used in the study for better understanding of the readers.

Response: We have elaborated risk factor identification methods. Lines: 144-151.

8. In figure 1, authors have mentioned 11 RNA fragments but in the figure only 10 RNA segments are visible, 8th RNA segment in all groups is not visible. Please justify this. It will be good if authors will mention about the encoded proteins of different RNA fragments in figure as well as in the discussion section.

Response: In figure 1, segments 8 and 9 are fused together while segment 7 is separate. So, it appears that there are 10 segments. The gene segments encoded proteins were mentioned in Figure 1 and also in the introduction.

9. In figure 2, label the name of gene (VP7 and VP4) along with product length.

Response: In figure 2, we have added the name of the genes with their product length. This has also been described in lines 161-162 and 166-167,

10. Author have stated two contradictory statements, Page 3, line No 3-4, and Page 12, line No 201-203, please clarify this.

Response: We did not find any contradictory statement in the mentioned places. However, we have rearranged lines 3-4 in page 3.

11. The pattern of cited references in the list is not consistent, please do the needful corrections.

Response: We have corrected the reference lists.

12. There are few grammatical and typographical mistakes in the manuscript, so authors should carefully read the manuscript and do the needful corrections.

Response: We have carefully read the revised manuscript and corrected the typographical mistakes. The grammatical mistakes were corrected by a native English speaking co-author.
