# Supplementary material for: Risk factors for bovine rotavirus infection and genotyping of bovine rotavirus in diarrheic calves in Bangladesh
Source: PLoS One. 2022 Feb 25;17(2):e0264577. doi: 10.1371/journal.pone.0264577 (PMC8880881; doi:10.1371/journal.pone.0264577)
Supplement: Attachment — Submitted filename: Response to reviewers.docx [file pone.0264577.s005.docx]

We thank all reviewers for their valuable comments to improve the manuscript further. The changes we made in the revised version were shown in blue font.

Reviewer #1: All previous comments have been answered except for minor language corrections and need of citation at one place. Please refer to details here.

line 128 - centrification - should be centrifugation.

Response: centrification was changed to centrifugation in line 128.

line 163 - Rephrase to "RT-PCR targeting the genes - VP7 and VP4 were used to detect BRV."

Response: Rephrased as suggested by the reviewer.

lines 216-7 - age is represented as months here while in previous sentences ages is mentioned in weeks. Please rectify.

Response: Age was converted to weeks according to the suggestion of a reviewer. However, we forgot to replace this in lines 216-217. Now, we have replaced months by weeks in lines 216-217.

line 232 - Correct "segemnts" to "segments".

Response: “Segemnts” was corrected to “segments” in line 232.

Line 238-41 - Rephrase to "Based on the subtype-specific RT-PCR and web-based analysis, among the 18 amplicons evaluated, 17 were BRV G6 (94.4%) and 1 BRV G10 (5.6%). This indicates that G6 is the predominant genotype."

Response: Rephrased as suggested by the reviewer in lines 238-241..

Line 345 - What percentage of the total land of the nation, do the 3 districts cover?

Response: It represents only 5% of Bangladesh. We have added this in lines 353-353..

Line 346 - Give reference of the "another study".

Response: We have added one reference in line 354.

Reviewer #2: Authors have not performed the suggested experiments and have also not properly modified the manuscript as per the suggestion. More so, they have not well responded to the comments.

Response: In fact, it is not clear what the reviewer means by “not performed the suggested experiments and have also not properly modified the manuscript as per the suggestion. The reviewer also mentioned that we did not respond properly to his comments”. As the reviewer did not make any specific comment, we are not able to address to the specific point in the revised manuscript. However, we have attached our response again to every comment of the reviewer in the first revision.

=====================================================================

Reviewer comments and response were in the first revision:

1. Authors should also discuss the impact of other factors particularly, season and sex in the abstract.

Response: Season and sex were not significantly associated with BRV infection. So we did not add them in the abstract.

2. In the introduction section, authors have discussed more about the genotypes of BRV; it will be good, if they will reduce this part and include a paragraph on the impact of different parameters (such as Herd size, colostrum feeding, colostrum timing and colostrum amount) on the rate of BRV infection in calves.

Response: We have reduced the introduction section in relation to the BRV genotypes and added one paragraph about the effect of herd size, colostrum feeding, timing and amount. Lines: 87-94.

3. If possible, authors should also examine the impact of other factors on BRV infection, which are also identified as one of the potential factors (such as Herd size, colostrum feeding, colostrum timing and colostrum amount) for BRV infection.

Response: Thank you very much for this comment. It could be valuable if these variables were included in the analysis. However, we don’t have data for these variables.

4. In this study, author found that female calves are more susceptible for BRV infection while studies have also shown that male calves are more susceptible for BRV infection, what could be the possible reason; please discuss this in to the discussion section.

Response: As mentioned in the response to a previous comment of the reviewer that sex was not statistically associated with BRV infection. So we did not describe it in the manuscript.

6. Discuss the principle of rapid detection kit BIO K 152 behind the detection of BRV in the materials and methods.

Response: The principle of rapid detection kit BIO K 152 was added in the materials and method section as suggested by the reviewer. Lines: 113-116.

7. Authors should elaborate the methods used in the study for better understanding of the readers.

Response: We have elaborated risk factor identification methods. Lines: 144-151.

8. In figure 1, authors have mentioned 11 RNA fragments but in the figure only 10 RNA segments are visible, 8th RNA segment in all groups is not visible. Please justify this. It will be good if authors will mention about the encoded proteins of different RNA fragments in figure as well as in the discussion section.

Response: In figure 1, segments 8 and 9 are fused together while segment 7 is separate. So, it appears that there are 10 segments. The gene segments encoded proteins were mentioned in Figure 1 and also in the introduction.

9. In figure 2, label the name of gene (VP7 and VP4) along with product length.

Response: In figure 2, we have added the name of the genes with their product length. This has also been described in lines 161-162 and 166-167,

10. Author have stated two contradictory statements, Page 3, line No 3-4, and Page 12, line No 201-203, please clarify this.

Response: We did not find any contradictory statement in the mentioned places. However, we have rearranged lines 3-4 in page 3.

11. The pattern of cited references in the list is not consistent, please do the needful corrections.

Response: We have corrected the reference lists.

12. There are few grammatical and typographical mistakes in the manuscript, so authors should carefully read the manuscript and do the needful corrections.

Response: We have carefully read the revised manuscript and corrected the typographical mistakes. The grammatical mistakes were corrected by a native English speaking co-author.

====================================================================

Reviewer #3: Comments:

1) In the present study authors focused only on three districts, what was the status of diarrheic calves in other districts of Bangladesh?

The status of BRV infection in diarrheic calves in other districts of Bangladesh was discussed in the discussion section of the first revision. We have highlighted those in lines: 293-301.

2) As authors said they used BRV rapid detection kit which only provides the information about the presence and absence of infection, what was the percentage of infection does any other method or technique to calculate the % of infection?

Response: Initially, we screened all samples by BRV rapid detection kit. The rapid test positive samples were further confirmed by PAGE. So, the percentage of infection according to the serial interpretation of both tests will be 15% (30/200). We have now added this result in the result section. Lines: 223-224.

3) In the present study authors find the G and P genotypes in diarrheic calves, if authors prefer the HRM (high resolution melting) analysis in control and infected calves to find out the SNP (single nucleotide polymorphism) which is responsible for the diarrheic condition?

Response: In our study, all the fecal samples were collected from diarrheic calves, showing symptoms of diarrhea. Sample was not taken from healthy cows. Therefore, comparison of Rotavirus genes between diarrheic cows and healthy cows was not possible, and SNP in VP7 and VP4 was not revealed in our study design.

4) In figure 2a lane no. four there is light amplified product, what represent this band??

Response: We consider that this band was nonspecific amplicon, because the size was a little larger than expected and faint. In the revised manuscript, additional description was inserted to legend of Fig.2 as follows (underlined). “……Lanes: 1-5, suspected diarrheic stool samples. Successful amplification of VP7 and VP4 genes is seen in lane  1, 3, 5 (a) and lane 1, 3, 4, 5 (b), respectively.”

Minor comments:

Line no.116,153,171,200,201,258,294 and 304 two-three words are combined ,this might be due to mac or windows please once check it.

Response: Sorry for this unintentional mistake. We have checked the manuscript and corrected all combined words.
