# Supplementary material for: Risk factors for bovine rotavirus infection and genotyping of bovine rotavirus in diarrheic calves in Bangladesh
Source: PLoS One. 2022 Feb 25;17(2):e0264577. doi: 10.1371/journal.pone.0264577 (PMC8880881; doi:10.1371/journal.pone.0264577)
Supplement: Attachment — Submitted filename: Reviewer comments.docx [file pone.0264577.s003.docx]

In the present study, Ahmed et al. has tried to identify G and P genotypes of bovine rotavirus (BRV) strains in diarrheic calves as well as the major risk factors of BRV infection. In this study, Ahmed et al. noticed the predominant role of G6P[11] genotype (94.4%), followed by G10P[11] (5.6 %) in BRV infection. Further, their experimental evidence suggested age and breed of claves as major risk factors for the BRV infection.

**Comments:**

**1.** Authors should also discuss the impact of other factors particularly, season and sex in the abstract.

**2.** In the introduction section, authors have discussed more about the genotypes of BRV; it will be good, if they will reduce this part and include a paragraph on the impact of different parameters (such as Herd size, colostrum feeding, colostrum timing and colostrum amount) on the rate of BRV infection in calves.

**3.** If possible, authors should also examine the impact of other factors on BRV infection, which are also identified as one of the potential factors (such as Herd size, colostrum feeding, colostrum timing and colostrum amount) for BRV infection.

**4.** In this study, author found that female calves are more susceptible for BRV infection while studies have also shown that male calves are more susceptible for BRV infection, what could be the possible reason; please discuss this in to the discussion section.

**6.** Discuss the principle of rapid detection kit BIO K 152 behind the detection of BRV in the materials and methods.

**7.** Authors should elaborate the methods used in the study for better understanding of the readers.

**8.** In figure 1, authors have mentioned 11 RNA fragments but in the figure only 10 RNA segments are visible, 8^th^ RNA segment in all groups is not visible. Please justify this. It will be good if authors will mention about the encoded proteins of different RNA fragments in figure as well as in the discussion section.

**9.** In figure 2, label the name of gene (VP7 and VP4) along with product length.

**10.** Author have stated two contradictory statements, Page 3, line No 3-4, and Page 12, line No 201-203, please clarify this.

**11.** The pattern of cited references in the list is not consistent, please do the needful corrections.

**12.** There are few grammatical and typographical mistakes in the manuscript, so authors should carefully read the manuscript and do the needful corrections.
